# Online Cognitive-Behavioral Programs for Women Living With Endometriosis: Protocol for a Scoping Review

**DOI:** 10.2196/75981

**Published:** 2025-12-08

**Authors:** Olivia S Parker, Sean R Locke, Kimberley L Gammage, Nancy C Gyurcsik, Danielle R Dunwoody

**Affiliations:** 1 Department of Kinesiology, Brock University, 1812 Sir Isaac Brock Way, St. Catharines, ON, L2S 3A1, Canada, 1 905-688-5550 ext 4958; 2 College of Kinesiology, University of Saskatchewan, Saskatoon, SK, Canada; 3 Department of Nursing, Brock University, St. Catharines, ON, Canada

**Keywords:** endometriosis, pain, health-related quality of life, online program, online intervention, behavior change techniques, PRISMA

## Abstract

**Background:**

Endometriosis is a chronic inflammatory condition experienced by approximately 10% of women worldwide. Women living with endometriosis experience multidimensional burdens often attributed to pain, fatigue, anxiety, and depression. Treatment patterns frequently focus on acute symptom management, while endometriosis is a lifelong condition. In addition, endometriosis-associated symptoms are complex, requiring multimodal treatment strategies. New complementary approaches for symptom management are needed, where further exploration of current online programs is a necessary next step. Online programs can be noninvasive and convenient, provide a sense of comfort, and are often more cost-effective than traditional in-person medicine. Mapping components within the online programs, using the behavior change ontologies, for women living with endometriosis is necessary for developing effective and long-lasting endometriosis management options.

**Objective:**

The aim of this scoping review is to examine the range and nature of online cognitive-behavioral programs created to manage endometriosis-associated symptoms and to identify key behavior change techniques (BCTs) within the online programs.

**Methods:**

We will conduct the proposed scoping review using the Arksey and O’Malley framework with an interpretive scoping review, consulting stakeholder methodology (6 stages) and referencing the Joanna Briggs Institute Evidence Synthesis Manual. The population-situation framework was used to develop our search strategy, focusing on women living with endometriosis (population) and online programs or online interventions (situation). The study must be a primary source of data, published in 2014 or later, the intervention must take place online and must seek long-term changes for symptom management. Original studies, including peer-reviewed and protocol studies, will be identified from Ovid MEDLINE, Ovid Embase, EBSCOhost CINAHL Complete, ProQuest Nursing and Allied Health Premium, EBSCOhost SPORTDiscus, and ProQuest PsycINFO. Preprints will be identified via the Web of Science Preprint Citation Index. We will also search for active clinical trials from multiple registries. EndNote (Clarivate) will be used for citation management, and Covidence (Veritas Health Innovation Ltd) software will be used for screening and extraction. Up to 5 reviewers will screen studies in Covidence using an inclusion checklist for title and abstract screening and then full-text review. Included studies will be coded to identify BCTs within the online intervention using the BCTs Taxonomy v1. Results will report on the characteristics of the included studies, the BCTs used in the online program or intervention, and the outcomes of the program. Results, including tables and the narrative, have been piloted prior to publication of this protocol using 4 studies that meet our criteria.

**Results:**

Data collection is scheduled to start in October of 2025, with results to be published by the Spring of 2026. This research is not directly funded.

**Conclusions:**

This review will collate information about online symptom management interventions for women living with endometriosis. Findings will identify gaps that can guide future intervention development.

## Introduction

Endometriosis is a chronic inflammatory and estrogen-dependent disorder in which tissue that mimics the endometrium of the uterus grows outside of the uterus in ectopic sites [1,2]. Endometriosis-associated inflammation can lead to a lower pain threshold via central sensitization and dysregulation of the hypothalamic-pituitary-adrenal axis [3,4]. It commonly affects women of reproductive age, with symptoms ranging widely from pelvic pain and fatigue to gastrointestinal issues, anxiety, and infertility [5,6].

Endometriosis negatively impacts quality of life (QoL), with many women experiencing emotional distress and social isolation, leading to feelings of hopelessness [6]. Endometriosis-associated pain, one of the most common symptoms, can be associated with neuropathic or nociplastic pain, which can vary in intensity [3] and is often exacerbated by stress and psychological factors (eg, anxiety) [7]. Women living with symptomatic endometriosis report worse mental health and day-to-day pain than women living with asymptomatic endometriosis [8]. The endometriosis health profile is a validated health-related QoL self-report measure often used to understand symptom burden on QoL [9]. Due to the wide array of symptoms, multimodal therapy is often required [5,6].

Common treatment options for pain include surgery, hormone therapies, and pain medications (eg, nonsteroidal anti-inflammatory drugs) [10], though more long-term, holistic options are needed. Nonmedical options have also shown promise in helping patients manage their endometriosis-related symptoms. Self-management strategies, such as diet changes [11] or stress reduction [12], have shown short-term benefits, but more research is needed to determine long-term effectiveness. Systematic reviews have concluded that research on physical activity is limited with poor intervention designs and mixed findings regarding improvements in endometriosis-associated symptoms [13,14].

Cognitive behavioral therapies have been shown to decrease pain sensation, improve depression and stress, normalize cortisol levels, and improve aspects of QoL (eg, emotional well-being, control, and supporting autonomy) in women living with endometriosis [15]. Intervention techniques, such as psychoeducation, goal-setting, problem-solving, coping strategies, relaxation training, stress management, and mindfulness approaches, have been used individually or in combination to help women manage their endometriosis [15-17]. Longer duration interventions and randomized controlled trials are needed to understand which cognitive-behavioral techniques are most beneficial and feasible. A scoping review can map the current literature in this area and identify beneficial behavior change techniques (BCTs) used within these interventions.

Since 2020, research involving online programs or interventions has greatly increased due to the worldwide restrictions implemented due to the COVID-19 pandemic [18]. Health care providers, such as nurses and psychologists, have provided digital health care services to their users, such as monitoring and consulting [18]. Online programs can be effective in reducing barriers associated with behavior change and participation. Specifically, for those with endometriosis, online intervention may reduce the stigma associated with seeing a specialist [19]. In the broader pelvic pain population, Seo and Seo [20] conducted a review on the effectiveness of digital health care for managing menstrual-related symptoms among reproductive-age women, reporting that digital and web-based interventions, such as counseling, tele-yoga, and internet-based cognitive behavioral therapies, are accessible and effective tools for managing menstrual symptoms [20].

For women living with symptomatic endometriosis, a single-session digital exercise intervention is effective at providing immediate pain relief [21]. In addition, short-duration online interventions aiming to improve QoL in women living with endometriosis have found improvements in QoL domains of control and powerlessness, emotional well-being, and social support [12,22]. There are active protocol studies involving an online program [19,23], where an in-depth investigation of online programs is necessary for future study designs for improving endometriosis-associated symptoms.

This scoping review aims to examine the range and nature of online cognitive-behavioral programs created to manage endometriosis-associated symptoms. This proposed review will collate identified psychological and behavioral techniques and map the extent of online management options available for women living with endometriosis. The BCT Taxonomy v1 [24] will provide a standardized language for reporting findings, which is a foundational step toward identifying common strategies that can be replicated or used in future intervention development. In addition, the review will summarize the endometriosis-associated symptoms investigated and the outcomes of the online interventions to help identify gaps in online interventions or programs for women living with endometriosis.

## Methods

### Study Design

This scoping review is guided by the Arksey and O’Malley framework [25], with an interpretive scoping review that consulted stakeholder methodology. The Joanna Briggs Institute Evidence Synthesis Manual will be used synergistically [26]. The data analysis process involves 5 stages, with a sixth describing stakeholder engagement. We will submit the Joanna Briggs Institute scoping review fillable checklist with the completed review (PRISMA-ScR [Preferred Reporting Items for Systematic Reviews and Meta-Analyses Extension for Scoping Reviews]; Checklist 1 [27,28]).

### Protocol

The protocol document was drafted using the Joanna Briggs Institute scoping review template [29] and PRISMA-P (Preferred Reporting Items for Systematic Review and Meta-Analysis Protocols) checklist [30]. We developed a comprehensive search strategy in collaboration with a Brock University Librarian, Elizabeth Yates. In addition, the Brock Library evidence synthesis review work plan document was completed [31] for the preparation of this protocol. The Consolidated Standards of Reporting Trials specific to reporting pain clinical trials [32] were referenced to ensure all criteria for optimizing pain-controlled trials reporting are represented accordingly in this scoping review.

### Stage 1: Identifying the Research Questions

We used the population-situation framework [33] to develop our research questions. We originally considered using the population, concept, and context framework but dismissed this because we aim to report on symptoms (context) that the online programs or interventions are targeting. The population-situation framework is used for descriptive and exploratory questions in the health care field. Our “concept one” comprises all women living with endometriosis (population), and our “concept two” comprises online programs or interventions (situation). Identifying concepts within the research question also assisted with developing the search strategy. Our overarching research question is, “ What online cognitive-behavioral programs or interventions are available to manage endometriosis-associated symptoms?” We have three subquestions:

1. What are the characteristics of the online intervention or program?
2. What BCTs are used in the study design?
3. What are the outcomes (eg, endometriosis-associated symptoms) of the interventions or programs?

The BCT Taxonomy v1 (93 distinct BCTs) [24] is a method for standardizing language, specifying, and interpreting the active components of an intervention designed to change behavior [34]. There are 16 groupings and 93 hierarchically clustered BCTs. Identifying BCTs in the proposed scoping review provides an agreed-upon language for reporting content and is extremely useful for identifying what components of the intervention themselves may facilitate change or not [35]. In addition, descriptions of behavior change interventions vary widely, making research replicability, evaluation, and implementation in policy and practice difficult, hindering the development of effective interventions [34] in which we aim to address these concerns for online programming interventions for women living with endometriosis. Eligibility will be determined with the following criteria (Textbox 1).

Textbox 1. **Inclusion and exclusion criteria.**

**Inclusion criteria**

- Must be published in English between 2014 and the present year due to a shift in technology-delivered interventions [18] while accounting for earlier research.
- The intervention or program must seek to improve endometriosis-associated symptoms (eg, health-related QoL, pain, sleep, anxiety, depression, and fatigue) or seek to improve at least 1 psychological outcome (eg, pain acceptance, pain catastrophizing, and illness acceptance) in humans.
- Intervention must be online, with details on the components of the online program.
- Must be a primary source of data.

**Exclusion criteria**

- If the aim of the program is a stand-alone pain relief intervention or program (eg, only hormonal, drug, and pharmaceutical programs) or is designed for biomedical pain management.
- If the program is for diagnosis or diagnostic techniques.
- If the publication is gray literature (eg, websites and pamphlets), a thesis or conference presentation, or a review, we aim to capture full-text research studies that require detail in the intervention design.

### Stage 2: Identifying Relevant Studies

Operational definitions were created to assist in data analysis (refer to Table 1 for operational definitions). The following 6 databases will be searched for peer-reviewed and protocol studies: Ovid MEDLINE, Ovid Embase, EBSCOhost CINAHL Complete, ProQuest Nursing and Allied Health Premium, EBSCOhost SPORTDiscus, and ProQuest PsycINFO. Preprints will be searched using the Web of Science Preprint Citation Index [36]. Active research will also be searched from the ClinicalTrials.gov [37] search engine, World Health Organization International Clinical Trials Registry Platform, European Clinical Trials Register, Australian New Zealand Clinical Trials Registry, and Cochrane Central Register of Controlled Trials. We are including a variety of study designs that meet our criteria, consistent with other scoping reviews [38], including all primary sources of data, experimental studies, observational studies, and qualitative studies (excluding synthesized evidence). We are excluding gray literature (eg, websites), conference presentations, book chapters, and reviews to be able to identify specific components (BCTs) of the online program or interventions through published full-text studies.

**Table 1.** Operational definitions.

Word	Definition
Synchronous program	Live and real-time with supervision done at a specific time.
Asynchronous program	Prerecorded and can be done without supervision at any time.
Online program	Any internet- or technology-delivered (asynchronous, synchronous, or both) program or intervention aimed at improving symptoms related to endometriosis or improving a psychological domain.
Online intervention	May be an experimental study (eg, randomized controlled trials and controlled trials), an observational study (eg, cohort or longitudinal, case control, cross-sectional, ecological), a qualitative (eg, focus groups, interviews), or a combination that is internet or technology delivered.
Characteristics of the study	This would include basic information on the publication, for example, author, year, country of publication, stage of research, and publication type.
Characteristic of the online program	This would include the aim of the program itself, the mode of delivery, the length of the program, the delivery type, the explanation of the program, the cost of the program, the barriers or facilitators of the program, and the referral from a physician who delivers the intervention.
Behavior change techniques (BCTs)	BCTs are the smallest, observable, and replicable content of a behavior change program or intervention that have the potential to bring about a change [34]. BCTs will need to be hand-coded for the program or intervention using the BCT Taxonomy v1 as a guide.
Outcomes of the program	Outcomes of the program will include measures, time points, data analysis, and main findings.

### Search Strategy

All women living with endometriosis (concept one) and online programs or online interventions (concept two) were placed into Boolean circles, where the mining of terms began. Ovid MEDLINE was used to develop the search strategy, considering subject headings, keywords, synonyms, related terms, and search syntaxes for both concepts. For example, in concept one, we want to capture all women living with endometriosis, regardless of age, stage, and location. Mining subject headings and keywords in Ovid MEDLINE found terms, such as endometriosis, endo, endometrioma, endometriomas, endometrioses, chronic pelvic pain, fibroids, adhesions, and lesions. Concept two was broken down further into alternative words for online programs and alternative words for online interventions. The refinement of keywords and subject headings was run multiple times via Ovid MEDLINE. This search strategy will be translated from Ovid MEDLINE to incorporate the search syntax used in each database or search engine. The search strategy export will be completed in 1 day by 2 or more members of the research team collaboratively in October 2025. Our final Ovid MEDLINE search strategy is shown in Table 2.

**Table 2.** Search strategy: Ovid MEDLINE example.

Concept^a^ and line number	Searches	Results
Population		
1	exp^b^ Endometriosis/	26,901
2	endometrio*^c^.tw.^d^	39,415
3	exp pelvic pain/	11,805
4	(pelvi* adj^e^ pain).tw.	12,054
5	1 or 2 or 3 or 4	57,711
Situation 1		
6	exp Video Recording/ or Telephone/	59,514
7	exp Therapeutics/ or Rehabilitation/ or Patient Education/	54,41,101
8	6 and 7	12,833
9	exp Digital health/	468
10	exp Internet-Based Intervention/	1398
11	exp Internet/	1,02,716
12	exp exercise/	2,61,848
13	11 and 12	1339
14	8 or 9 or 10 or 13	15,926
Situation 2		
15	(online* adj5 (program* or education* or train* or intervention* or treatment* or therap* or platform* or -based* or prevent* or manag* or health* or rehab* or exercise* or physical acitivi*)).tw.	45,762
16	(virtual* adj5 (reality* or program* or education* or train* or intervention* or treatment* or therap* or platform* or -based* or prevent* or manag* or health* or rehab* or exercise* or physical acitivi*)).tw.	43,344
17	(“web” adj5 (reality* or program* or education* or train* or intervention* or treatment* or therap* or platform* or -based* or prevent* or manag* or health* or rehab* or exercise* or physical acitivi*)).tw.	59,086
18	(video* adj5 (program* or education* or train* or intervention* or treatment* or therap* or platform* or -based* or prevent* or manag* or health* or rehab* or exercise* or physical acitivi*)).tw.	30,530
19	(“app” adj5 (program* or education* or train* or intervention* or treatment* or therap* or platform* or -based* or prevent* or manag* or health* or rehab* or exercise* or physical acitivi*)).tw.	10,022
20	(internet* adj5 (program* or education* or train* or intervention* or treatment* or therap* or platform* or -based* or prevent* or manag* or health* or rehab* or exercise* or physical acitivi*)).tw.	24,909
21	(phone* adj5 (program* or education* or train* or intervention* or treatment* or therap* or platform* or -based* or prevent* or manag* or health* or rehab* or exercise* or physical acitivi*)).tw.	8329
22	(tele* adj5 (program* or education* or train* or intervention* or treatment* or therap* or platform* or -based* or prevent* or manag* or health* or rehab* or exercise* or physical acitivi*)).tw.	42,534
23	(digital* adj5 (program* or education* or train* or intervention* or treatment* or therap* or platform* or -based* or prevent* or manag* or health* or rehab* or exercise* or physical acitivi*)).tw.	37,988
24	14 or 15 or 16 or 17 or 18 or 19 or 20 or 21 or 22 or 23	2,79,541
25	5 and 24	369

a Concept: developed from the overarching research question using the population-situation framework.

b expand (subject heading search).

c truncation (includes anything after).

d search title and abstract (keyword search)

e adjacent within the number of words.

### Stage 3: Study Selection

Stage 3 describes the process for selecting studies. EndNote (Clarivate) citation management software will be used to organize citations. We will export our search results (from the databases and other sources) into Covidence (Veritas Health Innovation Ltd) software [39] to facilitate the process of removing duplicate items and screening. Merging into Covidence will assist with removing some duplicates. During the screening of titles and abstracts, additional duplicates will be removed by the reviewer by selecting the duplicate study option. Reasons for inclusion or exclusion of studies can be added with notes in Covidence. The data extraction process will be presented in the form of the PRISMA (Preferred Reporting Items for Systematic Reviews and Meta-Analyses) flowchart.

Up to 5 reviewers will independently screen titles and abstracts in Covidence. Two reviewers will screen each title and abstract based on our inclusion and exclusion criteria (“yes,” “no,” or “maybe”). If there is a disagreement, a third reviewer will determine the final result. This process will occur again with the studies labeled “yes,” under full-text review, with the inclusion and exclusion criteria. Reasons for inclusion and exclusion will be documented and reported in the PRISMA flowchart. Following screening, all reviewers will perform a full-text review for final inclusion in the scoping review. The primary author, OSP, has prior experience using Covidence and completing the scoping review process [40].

### Stage 4: Charting the Data

Three reviewers will independently extract characteristics from the included studies into a Microsoft Excel file. Once completed independently, all extraction characteristics will be discussed together and merged. Row 1 in the Microsoft Excel document includes examples and a definition of each study characteristic to assist all reviewers. The Microsoft Excel file is provided in Multimedia Appendix 1. Extraction characteristics are shown in Textbox 2.

Textbox 2. **Extraction characteristics.**

- Characteristics of the study: author, year, country of publication, stage of research, publication type, purpose, aims, research questions and hypothesis, sample size, study population, inclusion and exclusion criteria, recruitment methods, comparator condition, support or reject research question or hypothesis, limitations, and implications.
- Characteristics of the online programs: the aim of the program, mode of delivery, length of the program, delivery type, explanation of the program, cost of the program, barriers and facilitators, referral from a physician, who delivers the BCT, grouping of BCT, and BCT used.
- Outcomes of the study: measures, time points, data analysis, and main findings.

### BCT Coding

All reviewers will undergo training to code using the BCTs Taxonomy v1 [24,34,41]. This will involve a tutorial consisting of independent readings [24,34], group discussion, and practice coding using the online training course, designed to teach coders how to extract BCT from research studies [41]. The online training provides the opportunity to practice coding BCT excerpts and obtain real-time feedback. During the coding process, all included full-text studies will be downloaded, and all coders will independently code BCTs throughout the whole study to avoid unintentionally missing BCTs. Most of the BCTs will be within the “Methods” and if there are appendices. The number corresponding to the BCTs Taxonomy v1 [24] will be directly coded in the document and highlighted, leaving an audit trail, and then reported in the Microsoft Excel file. After independent coding of all studies, BCTs will be discussed together for accuracy checking.

### Pilot

The Microsoft Excel document for charting the data has been piloted with 4 target studies [12,19,22,23] that met the inclusion criteria. The file was modified once to include a separate folder strictly for coding BCTs from the BCTs Taxonomy v1 [24]. Tables and the narrative of the results have also been piloted and modified to answer our research questions to provide a comprehensive, logical framework.

### Stage 5: Collating, Summarizing, and Reporting the Results

We will have multiple tables that will report on the characteristics of the included studies and the characteristics within the online programs or interventions to address our first subquestion. A table will present frequencies (percentages) of the number of studies using specific BCTs (BCT Taxonomy v1) [24], and a table for the outcomes of the online programs, including target measures related to endometriosis and relevant results.

For the narrative component of the results, a framework will be adopted to prioritize certain aspects of the literature to ensure we answer our research questions [25]. The narrative will have four main sections: (1) an overview of study characteristics, (2) the characteristics of the online programs, (3) the BCTs within the online programs, and (4) the measures and outcomes of the programs. Tables will be used as complementary to the narrative, where new data may be presented in one or the other.

The discussion will be interpretive and reflexive, identifying similarities and differences between studies when necessary [25]. In addition, we will identify the next steps for online interventions or programs for women living with endometriosis. This will be presented in the “Discussion” section. The consulted stakeholder methodology will be used, drawing on expertise from all members of the research team during the interpretation of findings and the write-up [42].

### Stage 6: Consultation and Stakeholder Engagement

The proposed scoping review is a foundational step within a larger research program to identify the gaps in developing online symptom management programs for women living with endometriosis. Using the consulted stakeholder methodology by engaging researchers, a librarian, and multiple reviewers combines different perspectives of knowledge and expertise [25,42]. Identifying BCTs involves group engagement and discussion, as the BCTs are interpretations of direct words from the components of the online programs. We aim to minimize interpretation bias by using the BCT Taxonomy v1 and engaging in reflexive discussions [24,34]. The results of this study will be discussed with women living with a diagnosis of endometriosis for additional feedback, as well as with a variety of endometriosis specialists.

### Dissemination

All stakeholders will be invited to an online presentation on the findings of this scoping review. Results will be published with open access, and findings will be presented at international conferences. Results will be shared with students in a guest lecture, including how to conduct a scoping review and the importance of research. In addition, research findings will inform and be discussed in the next phase of our research project.

## Results

The pilot was conducted in December of 2024. Data collection is scheduled to start in October 2025, with results to be published by the spring of 2026.

## Discussion

### Principal Findings

This study sought to describe the process for reviewing evidence regarding online interventions or programs aimed at improving endometriosis-associated symptoms. The BCT Taxonomy and newer ontologies [34] were developed to provide a common nomenclature to report interventions, which allows for future synthesis and knowledge accumulation. We anticipate that BCT groupings, such as feedback and monitoring, social support, and shaping knowledge, will be coded in the majority of included studies, as found in other reviews of online programs, which coded BCTs [43,44]. We anticipate that the majority of studies will be early-stage interventions with few randomized controlled trials, given the nascent nature of this research area. Given the ubiquity and continued advancement of digital technologies, the proposed review is positioned to provide an overview of the current state of online interventions for endometriosis symptom management and has the potential to direct future research by illuminating research gaps.

Generally, reviews and meta-analyses have quantified the burden of endometriosis on mental health and examined predictors of mental health. For example, those with endometriosis experience poorer mental health [45], including more symptoms of anxiety and depression [46]. Those with better emotion regulation generally report better mental health functioning [47]. While these reviews paint a clear picture of the poor mental health status often associated with the progression of endometriosis, evidence for the impact of effective and scalable interventions to improve symptom burden is scant.

Past reviews have examined the benefits of clinic-based therapy for pain and QoL [48] among those living with endometriosis. Gandy et al [49] conducted a systematic review and meta-analysis to assess the efficacy of internet-delivered cognitive- and behavioral-based interventions for adults living with chronic pain. Authors concluded that internet-delivered cognitive and behavioral interventions are efficacious and associated with greater clinical gains, including pain management outcomes [49]. Pain is often accompanied by endometriosis; however, the experiences and strategies to manage may differ between individuals with endometriosis and those with chronic back pain. Whether online programs can improve endometriosis symptom burden has yet to be explored.

Online delivery improves program reach and may be beneficial for teaching self-management skills that are critical [22]. There are available online programs currently being evaluated and refined that aim to improve symptoms for women living with endometriosis, specifically on health-related QoL and pain [12,19,23]. An endometriosis-specific scoping review will contribute to the broad understanding of strategies currently used to promote symptom management. This review will identify gaps in current intervention design and provide suggestions to guide future online symptom management interventions.

### Limitations

There is a risk of misinterpretation of coding BCTs within the studies that outline few methodological details. While this may require researcher interpretation, the structured BCT training increases our confidence in accurate coding. This review includes all types of intervention designs, including early-stage trials. Thus, we will be unable to determine whether online interventions are generally efficacious at improving symptom management for women living with endometriosis.

### Conclusions

This scoping review will examine the range and nature of online cognitive-behavioral programs created to manage endometriosis-associated symptoms. By identifying similarities and differences between the intervention designs using the BCT Taxonomy v1 [24], we can guide future research in intervention development to improve endometriosis-associated symptoms. We will seek to publish findings that are open access and create graphics that are shared via social media.

## Supplementary material

10.2196/75981 Multimedia Appendix 1 Data extraction file.

10.2196/75981 Checklist 1 PRISMA-ScR checklist.

## Abbreviations

BCT: behavior change techniques
PRISMA: Preferred Reporting Items for Systematic Reviews and Meta-Analyses
PRISMA-P: Preferred Reporting Items for Systematic Review and Meta-Analysis Protocols
PRISMA-ScR: Preferred Reporting Items for Systematic Reviews and Meta-Analyses Extension for Scoping Reviews
QoL: quality of life

## References

[1] Bulun SE, Yilmaz BD, Sison C (2019). Endometriosis. Endocr Rev.

[2] Leyland N, Casper R, Laberge P, Singh SS, SOGC (2010). Endometriosis: diagnosis and management. J Obstet Gynaecol Can.

[3] Mechsner S (2022). Endometriosis, an ongoing pain—step-by-step treatment. J Clin Med.

[4] Morotti M, Vincent K, Becker CM (2017). Mechanisms of pain in endometriosis. Eur J Obstet Gynecol Reprod Biol.

[5] Singh S, Soliman AM, Rahal Y (2020). Prevalence, symptomatic burden, and diagnosis of endometriosis in Canada: cross‐sectional survey of 30 000 women. J Obstet Gynaecol Can.

[6] Soliman AM, Singh S, Rahal Y (2020). Cross-sectional survey of the impact of endometriosis symptoms on health-related quality of life in Canadian women. J Obstet Gynaecol Can.

[7] Kalfas M, Chisari C, Windgassen S (2022). Psychosocial factors associated with pain and health-related quality of life in Endometriosis: a systematic review. Eur J Pain.

[8] Facchin F, Barbara G, Saita E (2015). Impact of endometriosis on quality of life and mental health: pelvic pain makes the difference. J Psychosom Obstet Gynaecol.

[9] Jones G, Kennedy S, Barnard A, Wong J, Jenkinson C (2001). Development of an endometriosis quality-of-life instrument: The Endometriosis Health Profile-30. Obstetrics & Gynecology.

[10] Singh S, Soliman AM, Rahal Y (2021). Treatment patterns of women with endometriosis in Canada. J Endometr Pelvic Pain Disord.

[11] Oszajca K, Adamus A (2024). Diet in prevention and treatment of endometriosis: current state of knowledge. Curr Nutr Rep.

[12] Miazga E, Starkman H, Skolnik E (2022). Virtual mindfulness therapy for the management of endometriosis chronic pelvic pain: a novel delivery platform to increase access to care. J Minim Invasive Gynecol.

[13] Evans S, Fernandez S, Olive L, Payne LA, Mikocka-Walus A (2019). Psychological and mind-body interventions for endometriosis: a systematic review. J Psychosom Res.

[14] Tennfjord MK, Gabrielsen R, Tellum T (2021). Effect of physical activity and exercise on endometriosis-associated symptoms: a systematic review. BMC Womens Health.

[15] Donatti L, Malvezzi H, Azevedo B de, Baracat EC, Podgaec S (2022). Cognitive behavioral therapy in endometriosis, psychological based intervention: a systematic review. Rev Bras Ginecol Obstet.

[16] Hansen S, Sverrisdóttir UÁ, Rudnicki M (2021). Impact of exercise on pain perception in women with endometriosis: a systematic review. Acta Obstet Gynecol Scand.

[17] Lunde CE, Wu Z, Reinecke A, Sieberg CB (2024). The application of cognitive behavioral therapy for adolescent patients with endometriosis: a topical review. Cogn Behav Pract.

[18] Vargo D, Zhu L, Benwell B, Yan Z (2021). Digital technology use during COVID‐19 pandemic: a rapid review. Hu Behav & Emerg Tech.

[19] Schubert K, Lohse J, Kalder M, Ziller V, Weise C (2022). Internet-based cognitive behavioral therapy for improving health-related quality of life in patients with endometriosis: study protocol for a randomized controlled trial. Trials.

[20] Seo MS, Seo BN (2025). Effectiveness of digital healthcare in managing menstrual symptoms: a systematic review. Womens Health Nurs.

[21] Lutfi M, Dalleck LC, Drummond C (2023). A single session of a digital health tool-delivered exercise intervention may provide immediate relief from pelvic pain in women with endometriosis: a pilot randomized controlled study. Int J Environ Res Public Health.

[22] Rohloff N, Rothenhöfer M, Götz T, Schäfer SD (2024). Observational pilot study on the influence of an app-based self-management program on the quality of life of women with endometriosis. Arch Gynecol Obstet.

[23] Escriva-Boulley G, Philip CA, Warembourg S (2023). Effects of a physical activity and endometriosis-based education program delivered by videoconference on endometriosis symptoms: the CRESCENDO program (inCRease physical Exercise and Sport to Combat ENDOmetriosis) protocol study. Trials.

[24] Michie S, Richardson M, Johnston M (2013). The behavior change technique taxonomy (v1) of 93 hierarchically clustered techniques: building an international consensus for the reporting of behavior change interventions. Ann Behav Med.

[25] Arksey H, O’Malley L (2005). Scoping studies: towards a methodological framework. Int J Social Research Methodology.

[26] Peters MDJ, Godfrey CM, Mcinerney P, Soares CB, Khalil H, Parker D (2015). The Joanna Briggs Institute reviewers’ manual 2015: methodology for JBI scoping reviews. https://reben.com.br/revista/wp-content/uploads/2020/10/Scoping.pdf.

[27] (2024). Preferred Reporting Items for Systematic Review and Metanalysis Extension for Scoping Reviews (PRISMA-ScR) fillable checklist. https://www.equator-network.org/wp-content/uploads/2018/09/PRISMA-ScR-Fillable-Checklist.pdf.

[28] Tricco AC, Lillie E, Zarin W (2018). PRISMA Extension for Scoping Reviews (PRISMA-ScR): checklist and explanation. Ann Intern Med.

[29] (2024). Resources. Joanna Briggs Institute.

[30] Shamseer L, Moher D, Clarke M (2015). Preferred Reporting Items for Systematic Review and Meta-Analysis Protocols (PRISMA-P) 2015: elaboration and explanation. BMJ.

[31] Hayden A, Premji Z, Yates E, Ash K (2022). Evidence synthesis review work plan. Brock University.

[32] Gewandter JS, Eisenach JC, Gross RA (2019). Checklist for the preparation and review of pain clinical trial publications: a pain-specific supplement to CONSORT. Pain Rep.

[33] (2024). Nursing. McGill.

[34] Marques MM, Wright AJ, Corker E (2023). The behaviour change technique ontology: transforming the behaviour change technique taxonomy v1. Wellcome Open Res.

[35] Cline A, Knowles N (2024). Identifying and applying behaviour change techniques. https://phwwhocc.co.uk/wp-content/uploads/2024/02/Identifying-and-Applying-Behaviour-Change-Techniques-1.pdf.

[36] Preprint citation index. Web of Science.

[37] ClinicalTrials.gov.

[38] Pham MT, Rajić A, Greig JD, Sargeant JM, Papadopoulos A, McEwen SA (2014). A scoping review of scoping reviews: advancing the approach and enhancing the consistency. Res Synth Methods.

[39] Covidence.

[40] Dagenais M, Parker O, Galway S, Gammage KL (2023). Online exercise programming among older adults: a scoping review. J Aging Phys Act.

[41] BCT Taxonomy.

[42] Buus N, Nygaard L, Berring LL (2022). Arksey and O’Malley’s consultation exercise in scoping reviews: a critical review. J Adv Nurs.

[43] van Vugt M, de Wit M, Cleijne W, Snoek FJ (2013). Use of behavioral change techniques in web-based self-management programs for type 2 diabetes patients: systematic review. J Med Internet Res.

[44] Webb TL, Joseph J, Yardley L, Michie S (2010). Using the internet to promote health behavior change: a systematic review and meta-analysis of the impact of theoretical basis, use of behavior change techniques, and mode of delivery on efficacy. J Med Internet Res.

[45] Wang Y, Li B, Zhou Y (2021). Does endometriosis disturb mental health and quality of life? A systematic review and meta-analysis. Gynecol Obstet Invest.

[46] van Barneveld E, Manders J, van Osch FHM (2022). Depression, anxiety, and correlating factors in endometriosis: a systematic review and meta-analysis. J Womens Health (Larchmt).

[47] Carvalho SA, Eulálio I, Guiomar R (2025). A systematic review and meta-analysis of the relationship between emotion regulation, pain, depressive symptoms and quality of life in women with endometriosis. J Psychosom Res.

[48] Abril‐Coello R, Correyero‐León M, Ceballos‐Laita L, Jiménez‐Barrio S (2023). Benefits of physical therapy in improving quality of life and pain associated with endometriosis: a systematic review and meta‐analysis. Intl J Gynecology & Obste.

[49] Gandy M, Pang STY, Scott AJ (2022). Internet-delivered cognitive and behavioural based interventions for adults with chronic pain: a systematic review and meta-analysis of randomized controlled trials. Pain.

